# Assessment of the Efficacy of Platelet-Rich Plasma in the Treatment of Traumatic Canine Fractures

**DOI:** 10.3390/ijms20051075

**Published:** 2019-03-01

**Authors:** Sergio López, José M. Vilar, Joaquín J. Sopena, Elena Damià, Deborah Chicharro, José M. Carrillo, Belén Cuervo, Mónica Rubio

**Affiliations:** 1 Animal Pathology Department, Instituto Universitario de Investigaciones Biomédicas y Universitarias, Universidad de Las Palmas de Gran Canaria, 35416 Trasmontaña S/N, Arucas, Spain; sergiolopezbarbeta@gmail.com (S.L.); jose.vilar@ulpgc.es (J.M.V.); 2 Bioregenerative Medicine and Applied Surgery Research Group, Animal Medicine and Surgery Department, Veterinary Faculty, Universidad Cardenal Herrera-CEU, CEU Universities, 46115 Valencia, Spain; jsopena@uchceu.es (J.J.S.); elena.damia@uchceu.es (E.D.); debora.chicharro@uchceu.es (D.C.); belen.cuervo@uchceu.es (B.C.); mrubio@uchceu.es (M.R.); 3 García Cugat Foundation CEU-UCH Chair of Medicine and Regenerative Surgery, 08006 Barcelona, Spain

**Keywords:** PRGF, Carprofen, dog, fracture, bone healing

## Abstract

The role of platelet-rich plasma (PRP) in promoting the healing of bone fractures has not yet been clearly stated. The aim of this prospective clinical study was to evaluate the effectiveness of plasma rich in growth factors (PRGF, a PRP derivate) in the treatment of naturally-occurring bone fractures in dogs. With this objective, sixty-five dogs with radius/ulna or tibia/fibula bone fractures were randomly divided into two groups (PRGF and saline solution (SS) groups) and checked at days 0, 7, 14, 21, 28, 35, 42, 49, 56, 60, 63, 70, 120, and 180. All the fractures were treated with an external skeletal fixation, and pain was controlled with Carprofen. Healing was evaluated by physical examination, limb function, radiography, and by a Likert-type owner satisfaction questionnaire. A faster fracture healing was observed in the PRGF group, with statistically significant differences with respect to the SS group. Swelling at the fracture site was significantly greater at day 14 and 28 in animals injected with PRGF, and more pain on palpation was found in the area at day 28. The injection of PRGF in acute bone fractures accelerates bone healing.

## 1. Introduction

Plasma-rich growth factors (PRGF) are currently being used to promote bone healing in reconstructive surgeries [1,2,3]. In canine models, several experimental studies have published the effect of this platelet rich plasma derivate in osteoarthritis with differing results [4,5,6,7]. Platelets are very important in the wound healing process [1]; they rapidly arrive at the wound side and begin the coagulation process. In addition, they release multiple wound-healing growth factors and cytokines within 10 min [1,2,3]. Platelets are viable for seven days and will continue to release growth factors into the tissue during this time [8].

The use of PRGF is based on the assumption that higher platelet concentrations release significant quantities of growth factors, which aids in bone healing [9,10,11]. Specifically, growth factors are thought to be a contributing factor in bone regeneration and in increasing vascularization, which are vital features of the bone-healing process [6].

Treatments with PRGF have given excellent clinical results in oral and maxillofacial surgery in humans [9,12], and in bone and cartilage healing in animal studies [7,13,14]. Growth factors have also been used in the treatment of large wounds and skin defects in burn patients [15,16,17]. However, some controversial results can be found in the cited literature; therefore, the effectiveness of this technique requires further research.

To the authors’ knowledge, articles discussing fresh fractures and delayed fracture healing are very scarce [18,19]. In the veterinary field, no publications were found regarding the use of PRGF in fractures.

In this study, the dogs used were clinical patients, but also clinical animal models. In the present study, we hypothesize that treating canine bone fractures with PRGF would accelerate bone healing. Thus, the aim of this clinical trial is to evaluate the use of PRGF in the treatment of naturally occurring bone fractures in dogs.

## 2. Results

A total of 68 dogs were initially evaluated; however, only 43 met the necessary requirements to be included in the study.

The dogs were randomly assigned to either PGRF or SS groups. Twenty dogs were included in the PRGF group (47%) and 23 in the SS group (53%). The results for each dog belonging to either the PRGF or the SS groups are summarized in their respective tables (Table 1 and Table 2).

The mean weight for each group was 16.27 kg for the PRGF group and 13.07 kg for the SS group. Mean age was 40.85 and 57.17 months, respectively, with no statistical differences between groups in these parameters (*p* ≥ 0.08).

During the study, all the animals received Carprofen as a rescue analgesia at least one time during the first seven days except for 2 and 4 patients in the PRGF and SS groups, respectively, with no statistical differences between groups (*p* ≥ 0.05).

**Figure 1:**
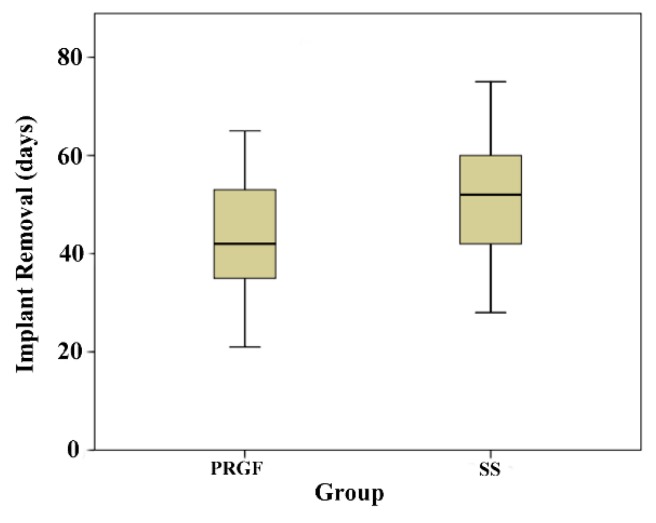
Boxplot corresponding to the days of implant removal for both PRGF and SS groups. Mean time was significantly higher in the SS group.

The time (mean ± SD) for implant removal was 41.3 ± 11.73 days in the PRGF group and 49 ± 12.12 days in the SS group. This difference was statistically significant (*p* = 0.03) (Figure 1). 

The time when full weight support was detected was 22.1 ± 13.64 days and 25.47 ± 14.9 days in the PRGF and SS groups, respectively; however, this difference was not statistically significant (*p* = 0.45). All animals were sound within six months post-surgery. 

Swelling in the fracture site was present in both groups up to day 14 without statistically significant differences between the groups. Between days 14 and 28, swelling was still present in the PRGF group (*p* < 0.048).

The joint movement evaluation showed almost 100% joint mobility without differences between groups in any of the checking periods.

The evaluation of pain on palpation showed statistically significant differences at day 28 between groups, where pain was still present in the PRGF group (*p* = 0.041).

No significant differences were found in the assessment of owner satisfaction at implant removal, with a satisfaction between 4 (24% in PRGF, 25% in SS) and 5 (76% in PRGF, 75% in SS). 

Complications were recorded. One dog suffered gastroenteritis, and three dogs had pins become loose in the PRGF group. The same number of complications occurred in SS group (Table 1 and Table 2).

## 3. Discussion

In the present study, the beneficial effect of PRGF in acute ulna/radius and tibia/fibula fracture healing has been proven, achieving a faster healing compared with controls. However, in all cases, a primary and non-complicated healing was present. 

To the authors’ knowledge, there is no published clinical research discussing the use of PRGF in fractures in a canine model. Experimentally, some studies proved there was faster bone regeneration when PRGF or other autologous platelet concentrates were applied [20,21]. In human medicine, there was only one clinical study evaluating the healing of fresh fractures using PRGF with no positive effect [18]. On the contrary, a clinical case with a delayed union fracture treated with autologous PRGF showed a favorable healing and concluded to be a safe technology for patients [19].

PRGF has also been used by other authors in combination with other therapeutics, showing positive results. Ya-dong Zhang et al. [22] proved that the use of PRGF combined with a degradable bioactive borate glass promotes functional bone repair. On the other hand, other authors [4] found no effect of PRGF on non-grafted implants in dogs; nevertheless, we cannot compare these results with our study because a different process was used to obtain the PRGF: using thrombin (100U/mL) to stimulate growth factor release rather than calcium chloride.

It is known that Carprofen is suitable alone or in combination with other NSAIDs for the control of pain and swelling in dogs [23,24]. Gastrointestinal inflammation and ulceration are among the most common side defects reported in the literature [25]. In our study, there were only two animals with gastroenteritis, and they responded positively to the conventional treatment. 

In the present study, it has been observed that the surgical application of PRGF at the fracture site is associated with increased swelling and oedema during the first days, probably due to the activation of angiogenesis and cell activation [26]. The enhancement of the arrival and formation of blood vessels increases heat, pain, and redness of the area. This swelling associated with oedema has been effectively treated with oral Carprofen. 

In any case, increased swelling did not affect the animals’ gait nor the functional ability of the joint. In this sense, some papers reported the inhibitory effect of interleukins, which may be attributed to PRP [27]. This effect may be related to a reduction of acute pain in the fracture site, even though the activation of angiogenesis may cause an increased perception of discomfort and inflammation [26]. Thus, even if the application of PRGF increases oedema and swelling on the area, the limb’s function was minimally affected during the first days. 

Good results have been obtained using PRP to accelerate bone fusion [28]. In our case, the group receiving the PRGF injection presented an earlier implant removal, which is in agreement with those who state that chemotactic and mitogenic effect on mesenchymal cells (stem cells) and osteoblasts accelerate bone healing [29,30]. 

A rapid return to functionality is crucial for quick and correct healing; when the limb bears weight, a transmission of forces takes place that stimulates osteoinduction. Likewise, early activity boosts vascularization and avoids muscle atrophy, which are factors that clearly activate bone healing. Moreover, the Carprofen helped to control pain and acute swelling at the fracture site, facilitating an earlier return to functionality [29]. This shows that swelling control and post-surgical analgesia are fundamental for early functionality of the affected limb and represent an important parameter to be assessed by the pet owners. 

Regarding external fixation, all the animals showed limb weight bearing 48 h after surgery. Very few complications arose in relation to the use of external skeletal fixation. One animal presented a secondary infection, which is a usual side effect, and only six animals presented pin loosening. Other studies show a larger number of cases presenting pin loosening as the most frequent complication [31].

The present study has three main limitations. First, the use of dogs with a wide weight range potentially limited results that are more accurate. A narrow weight range could provide more reliable and accurate results, at least for a specific weight range. Second, a biomechanical analysis of gait could provide full objective results regarding limb function. Third, statistical analysis of the variable “swelling at the fracture site” could provide more accurate results if it is considered a continuous variable instead of categorical, avoiding detection, performance, and reporting biases; however, the presence of hematoma or callous formation at the fracture site could potentially hinder precise measurements.

## 4. Materials and Methods

A multicentric study was designed and formed by four surgeons in four different veterinary clinical centers. 

### 4.1. Animal Model 

A total of 68 dogs were evaluated. The follow-up of the animals took place until six months after treatment. The inclusion criteria required the presence of a fresh, single, closed fracture and the absence of significant muscular soft tissue damage or abrasions. 

The exclusion criteria for the present study were the following:

- Animals presenting concurrent systemic disease (*Leishmania* spp., *Ehrlichia* spp., etc.).
- Animals with hematological disorders.
- Animals with multiple fractures.
- Animals with internal lesions due to traumatism.
- Animals with open fractures or with significant damage to the surrounding soft-tissue.
- Animals with a significant weight loss or functional disabilities due to the treatment or other non-related causes.
- Animals needing different concurrent fixation methods due to the nature or clinical features of the fracture.

Fractures were classified according to the affected bone. In order to acquire similar healing conditions during the study, only tibia/fibula and radius/ulna fractures were included because of their poor vascularization due to their small surrounding muscular mass. The individual data of each dog for the PGRF and SS groups are summarized in Table 1 and Table 2, respectively. 

### 4.2. Fracture Treatment

All fractures were treated with conventional open or closed reduction and external fixation. The external skeletal fixation configuration frame was the most appropriate for each fracture, using type IIa or type IIb [32]. In all cases smooth pins of different diameters, connecting bars, and Meynard clamps were used.

After an initial clinical examination, animals were randomly assigned to one of the following groups depending on the treatment received:

- PRGF group: A single infiltration of PRGF in the fracture site during the surgery.
- SS group: A single infiltration of saline solution in the fracture site during the surgery.

All groups were treated with morphine (0.2 mg IM every 6 h), and Carprofen 4 mg/kg IV (Rimadyl^®^, Zoetis^®^, Spain) for 24 h. Cephalexin was administered as a post-surgery antibiotic. 

After 24 h, the owners were allowed to give Carprofen (4 mg/kg/day) as a rescue analgesic if their pet presented clear signs of distress or discomfort. This fact should be reported during the clinical follow-up.

### 4.3. PRGF Preparation

For the present study, the extraction, isolation, activation, and administration model of the PRGF was standardized in all clinics following Anitua´s technique [9]. Briefly, 20 mL of blood were aseptically collected in four 4.5 mL citrate tubes, then centrifuged during 8 min at 460 G. Care has to be taken to avoid the buffy coat. Before the infiltration, the PRGF was activated with 5% of its volume with 10% calcium chloride. This obtained PRP derivate is enriched in platelets 2-fold over peripheral blood and less than 0.2 leucocytes × 10^6^/mL.

### 4.4. Evaluation

The limb function was evaluated on days 0 (pre-surgery), 7, 14, 21, 28, 60, 120, and 180 after the treatment began. This parameter was assessed by the same researcher evaluating animals when standing (1: weight-bearing; 2: no weight-bearing; or 3: no limb support), by observing swelling on the fracture site (0: presence or 1: absence), pain on palpation (0: presence or 1: absence), and joint movement (1: <40%; 2: 40–70%; 3: 70–90%; or 4: >90%). 

The same radiologist, unaware of the group of treatment, patient, and surgeon involved, examined all radiographs. Each radiograph was evaluated by a stage score of 1–5 points (1: not visible callus formation; 2: barely visible callus formation; 3: scattered, not homogeneous callus; 4: uniform, mature callus formation; 5: very active, hyperthrophic callus formation). Radiographical examination started for each dog at day 21 and for every two weeks thereafter until the animal reached stage 2; beyond this period, radiographs were taken weekly, coinciding with the checkpoints for the other parameters. When a final score of 4/5 was achieved, implant removal was performed and recorded. The researcher who performed the evaluation of limbs and who read the radiographies were blind to the given treatment (PGRF or SS).

The use of the rescue analgesic and the presence of side effects were registered by the owner. The level of owner satisfaction with the clinical outcome of their pets during the first 28 days and at implant removal was evaluated with the following questionnaire referring to the level of satisfaction measured with a Likert-type scale (Table 3).

### 4.5. Statistical Analysis

Statistical analysis was performed with the computer program SPSS 18^®^ for Windows^®^ (IBM Co., Chicago, IL, USA). A value of *p* < 0.05 was considered statistically significant. The descriptive study of the population was shown as the mean ± SD. To determine the differences between the groups for non-categorical variables (weight, age, and total doses of Carprofen), a Kruskal–Wallis and Mann–Whitney test was done. To determine the effect of PRGF on implant removal time, a Kaplan–Meier curve and a log-rank test were used. The impact evaluation of total doses of Carprofen, age, weight, and bone fractured, within time to implant removal, a multivariable analysis was made using a Cox regression. Categorical variables (evaluation when walking, evaluation when standing, swelling, pain on palpation, joint movement, use of the recue analgesic, owner satisfaction, and presence of side effects) were assessed using crosstabs with chi square, contingency coefficient, or the Fisher’s exact test used when necessary in each variable.

The experimental procedure was approved by the ethics committee of the Research Institute in Biomedical and Health Sciences (ULPGC, Spain). The owners were informed about the aims of the study, and a written consent was required before including their pets in the study.

## 5. Conclusions

The use of PRGF for bone repair accelerates fracture consolidation and simultaneously promoted healing, achieving clearly shorter implant removal times. 

## Figures and Tables

**Table 1:** Individual data and main results for PRGF group.

Dog #	Breed	Gender	Weight (kg)	Age (months)	Fracture L	Configuration	Weight Support	Time I Removal (days) *	Complications	Analgesia
1	G DANE	M	53	11	U/R	TYPE IIB 2X3	7	35 (39)	NO	Y
2	CROSSBREED	F	4	24	U/R	TYPE IIB 1X2	7	42 (45)	NO	N
3	CROSSBREED	M	17,8	36	U/R	TYPE IIB 2x4	21	28 (32)	NO	Y
4	CROSSBREED	F	8,5	96	U/R	TYPE IIB 2X3	14	56 (60)	GE	Y
5	CROSSBREED	F	4	3	T/F	TYPE IIB 2X3	7	21 (21)	NO	Y
6	CROSSBREED	M	4	36	U/R	TYPE IIB 2X3	28	63 (63)	NO	Y
7	CROSSBREED	M	7	48	T/F	TYPE IIB 2X3	21	35 (40)	PL	Y
8	CROSSBREED	F	6	6	T/F	TYPE IIB	21	28 (30)	NO	Y
9	CROSSBREED	F	2,3	60	U/R	TYPE IIB 2X3	21	42 (42)	PL	Y
10	CROSSBREED	F	22	12	U/R	TYPE IIB 2X3	14	35 (36)	NO	Y
11	CROSSBREED	F	72	20	U/R	TYPE IIB 2X3	60	56 (56)	NO	Y
12	CROSSBREED	M	6,3	48	U/R	TYPE IIB 2X3	60	42 (45)	NO	Y
13	CROSSBREED	M	4	12	U/R	TYPE IIB 2X3	7	56 (56)	NO	Y
14	BELG SHEPH	F	16	96	U/R	TYPE IIB 2X3	21	49 (49)	PL	Y
15	CROSSBREED	M	4,5	24	U/R	TYPE IIB 2X3	21	28 (30)	NO	Y
16	PODENCO	M	22	56	U/R	TYPE IIB 2X3	28	28 (28)	NO	Y
17	CROSSBREED	M	6	70	U/R	TYPE IIB 2X3	21	63 (65)	NO	Y
18	RAT VAL	F	2	24	U/R	TYPE IIB 2X3	21	49 (50)	NO	Y
19	MASTIFF	M	52	36	T/F	TYPE IIB 2X3	28	35 (40)	NO	Y
20	CROSSBREED	M	12	99	U/R	TYPE IIB 2X3	14	35 (35)	NO	N

* The first number references the checking day when stage 4/5 was reached radiographically and the implant was ready for removal; the number in parenthesis refers to the day the implant was removed. RAT VAL: Ratonero Valenciano.

**Table 2:** Individual data and main results for SS group.

Dog #	Breed	Gender	Weight (kg)	Age (months)	Fracture L	Configuration	Weight Support	Time I Removal (days) *	Complications	Analgesia
1	SIB HUSK	M	27	15	U/R	TYPE IIB	60	63 (69)	PL	Y
2	RAT VAL	F	1,7	6	U/R	TYPE IIB 1X2	21	35 (35)	NO	N
3	CROSSBREED	F	5,5	12	T/F	TYPE IIB1.5X2	7	28 (34)	NO	N
4	CROSSBREED	F	5,5	12	U/R	TYPE IIB1.5X2	7	28 (31)	PL	N
5	AM STAFFORD	M	30	72	U/R	TYPE IIB 2X3	21	35 (36)	NO	Y
6	CROSSBREED	M	4,5	60	U/R	TYPE IIB 2X3	7	56 (58)	NO	Y
7	CROSSBREED	M	20	72	U/R	TYPE IIB 2X3	21	70 (75)	NO	Y
8	GRIFFON	M	15	50	U/R	TYPE IIB 2X3	28	42 (42)	NO	Y
9	GER SHEPH	F	34	70	T/F	TYPE IIB 2X3	21	42 (44)	NO	Y
10	W HIGH W TERR	F	5,6	48	U/R	TYPE IIB 2X3	21	42 (42)	GE	Y
11	CROSSBREED	M	18	48	U/R	TYPE IIB 2X3	21	42 (45)	NO	Y
12	CROSSBREED	F	12	60	T/F	TYPE IIB 2X3	21	42 (45)	NO	Y
13	CROSSBREED	F	3	24	U/R	TYPE IIB 2X3	21	56 (57)	NO	Y
14	MALTESE	F	9	192	T/F	TYPE IIB 2X3	60	56 (60)	NO	Y
15	RAT VAL	F	5	111	T/F	TYPE IIB	21	63 (68)	NO	Y
16	BELG SHEPH	M	34	86	T/F	TYPE IIB	28	63 (65)	NO	N
17	BELG SHEPH	F	9	20	T/F	TYPE IIB 2X3	28	63 (64)	NO	Y
18	YORKSHIRE	F	1,5	35	U/R	TYPE IIB 2X3	21	35 (38)	PL	Y
19	POODLE	F	8	122	U/R	TYPE IIB 2X3	21	49 (50)	NO	Y
20	CROSSBREED	M	25	75	U/R	TYPE IIB 2X3	21	56 (60)	NO	Y
21	DALMATIAN	M	22	46	T/F	TYPE IIB 2X3	21	49 (52)	NO	Y
22	YORKSHIRE	F	1,5	24	U/R	TYPE IIB 2X3	60	56 (60)	NO	Y
23	CROSSBREED	M	4	55	U/R	TYPE IIB 2X3	28	56 (60)	NO	Y

* The first number references the checking day when stage 4/5 was radiographically reached and the implant was ready for removal; the number in parenthesis refers to the day the implant was removed. SIB HUSK: Siberian Husky; RAT VAL: Ratonero Valenciano; AM STAFFORD: American Staffordshire Terrier; GER SHEPH: German Shepherd; W HIGH W TERR: West Highland White Terrier; BELG SHEPH: Belgian Shepherd.

**Table 3:** Likert-type questionnaire of satisfaction for dog owners at time of implant removal.

**How do you consider the lameness of (name of the pet) has progressed?**				
Excellent	Good	Average	Fair	Poor
5	4	3	2	1
**Do you think the treatment given to (name of the pet) has been effective?**				
Strongly agree	Agree	Neutral	Disagree	Strongly disagree
**How Do You Think (Name of the Pet) has Responded to the Treatment?**				
Excellent	Good	Average	Fair	Poor
5	4	3	2	1

## Abbreviations

PRGF	Plasma rich in growth factors
SS	Saline solution
Fracture L	Fracture location
U/R	Ulna/radius
T/F	Tibia/fibula
Time I removal	Time for implant removal
GE	Gastroenteritis
PL	Pin loosening
Y	Yes
N	No
